# A genomic and phenotypic investigation of pigeon-adaptive *Salmonella*

**DOI:** 10.1371/journal.ppat.1012992

**Published:** 2025-03-17

**Authors:** Zining Wang, Zenghai Jiang, Qianzhe Cao, Chenghao Jia, Haiyang Zhou, Chenghu Huang, Linlin Huang, Yingying Huang, Yan Li, Min Yue

**Affiliations:** 1 Key Laboratory of Systems Health Science of Zhejiang Province, School of Life Science, Hangzhou Institute for Advanced Study, University of Chinese Academy of Sciences, Hangzhou, China; 2 Department of Veterinary Medicine, College of Animal Sciences, Zhejiang University, Hangzhou, China; 3 Hainan Institute of Zhejiang University, Sanya, China; 4 Department of Anesthesiology and Surgical Intensive Care Unit, Xinhua Hospital, School of Medicine and School of Biomedical Engineering and State Key Laboratory of Oncogenes and Related Genes, Institute for Personalized Medicine, Shanghai Jiao Tong University, Shanghai, China; 5 College of Veterinary Medicine, Henan University of Animal Husbandry and Economy, Zhengzhou, China; 6 State Key Laboratory for Diagnosis and Treatment of Infectious Diseases, National Clinical Research Center for Infectious Diseases, National Medical Center for Infectious Diseases, The First Affiliated Hospital, College of Medicine, Zhejiang University, Hangzhou, China; University of California Davis School of Medicine, UNITED STATES OF AMERICA

## Abstract

*Salmonella*, a significant threat to public safety, inflicts substantial economic losses on the poultry industry. The unique “parental feeding” breeding model of pigeon farms, against the “all-in & all-out” biosecurity strategy, makes them susceptible to *Salmonella* infections and subsequent outbreaks of pigeon paratyphoid. This study initially studied three pigeon paratyphoid outbreak incidents in Henan, China, in which 53 strains of pigeon-origin *Salmonella* Typhimurium (STM) were identified. Whole-genome sequencing (WGS) and antimicrobial-resistant profile analysis revealed that the three outbreaks were caused by distinct STM clones (ST128-DT2, ST19-DT99). Global phylogenetic analysis suggested that the United States is a possible origin, indicating a risk of intercontinental transmission via pigeon eggs. Further bacterial virulence and invasion assays, including *in vitro* and *in vivo* assays, revealed that pigeon-host-adaptive STM, compared to broad-host-range STM, carried fewer resistance genes, exhibited higher invasion indices and pseudogene levels, displayed a non-rdar (red dry and rough) phenotype, and had strong biofilm formation capability. Additionally, they showed reduced virulence and invasiveness in mice but a pigeon-adaptive feature in cogent models. The collective results support the host adaptation for pigeons among DT2 and DT99 phage-type isolates.

## Author summary

*Salmonella* is one of the four leading causes of diarrheal disease globally. The severity of the disease is contingent upon host factors and the specific serovars. *Salmonella* is a pervasive pathogen in domesticated and wild animals, particularly prevalent in poultry and mammals. It can disseminate throughout the food chain, from animal feed and primary production to the consumer level. The severe devastation of paratyphoid outbreaks in broiler pigeon farming and the widespread use of WGS technology have facilitated more reliable investigation into the evolution of host adaption of STM to pigeons and the traceability of global spread. The findings of our study indicate that all pathogenic isolates of three pigeon paratyphoid outbreaks in Henan, China, were likely transmitted from the United States. Both ST128-DT2 and ST19-DT99 isolates were historically considered pigeon-adaptive origin, while few studies investigated them from both mammalian and bird models. Here, we have employed a combination of comparative genomic approaches, as well as *in vivo* and *in vitro* assays, to investigate the pathobiology of two pigeon-adaptive types. Our collective findings demonstrate both types exhibiting host-adaptive towards pigeons. Importantly, we also find an ST128-DT2 lineage isolate showing a higher pigeon-tropism than the ST19-DT99 isolate. Nevertheless, our data offers valuable insights that could advance pigeon-adaptive *Salmonella* from genomic epidemiology to new biological traits, therefore guiding the policy for preventing paratyphoid fever outbreaks among pigeons.

## Introduction

*Salmonella*, a genus of rod-shaped, Gram-negative bacteria, is a prominent causative agent of foodborne illnesses worldwide[1]. These pathogens are responsible for a significant burden of gastroenteritis, typhoid fever, and paratyphoid fever, leading to considerable morbidity and mortality, especially in developing countries[2–4]. The Centers for Disease Control and Prevention (CDC) estimate that *Salmonella* infections cause approximately 1.35 million illnesses, 26,500 hospitalizations, and 420 deaths annually in the United States alone[5]. The pathogenicity of *Salmonella* is attributed to its ability to invade and replicate within host cells, evading the immune response through a sophisticated array of virulence factors. These include the Type III secretion system, which injects effector proteins into host cells to manipulate cellular processes, and the production of endotoxins that induce inflammation and tissue damage[6]. Furthermore, the emergence of multidrug-resistant *Salmonella* strains poses a substantial challenge to public health, complicating treatment options and exacerbating the spread of infections[7–9].

China is the country with the leading global poultry production due to its considerably large consumer market all over the world[10]. Squab, a young domestic pigeon under four weeks, has attracted increasingly consumers who pay attention to a healthy diet due to its balanced and rich nutritional value and good palatability. Nutritional research on squabs has been enriching gradually as well[11,12]. The wide range of activities and the large wild population make pigeons an important vector for the spread of epidemics and a potential intermediate host for a variety of zoonotic diseases[13,14]. Pigeon paratyphoid is a bacterial disease mostly in squabs caused by *Salmonella*, posing a potential threat to broiler pigeon farming and public health[15]. Literature studies have shown that paratyphoid fever is widespread in pigeon flocks in several countries, including Egypt[16], UK[17], Brazil[18], Italy[19], Norway[20], Belgium[21], Germany[22], Japan[23], Poland[24] and China[25].

In addition, as a significant threat to the domesticated poultry industry, *Salmonella* is a pathogen with significant zoonotic potential, which circulates among food animals and humans via the foodborne chain. Certain *Salmonella* serovar or their variant could lead to a host-adaptative stage, even restricting it to a particular host group[26]. A growing number of these pantropic *Salmonella* strains have exhibited specific host adaptations over time[27]. For example, *Salmonella* Typhimurium (STM) has shown host adaptation in various avian species, including phage types DT2 and DT99, which have adapted to pigeons[28–30]. The essence of host specificity changes lies in the immune evasion mechanisms of *Salmonella*, with pseudogenes representing the non-functional remnants of gene families that have been formed during the course of pathoadaptation, and single nucleotide polymorphisms (SNPs) being considered as causes for these host adaptations[31–33]. Emerging infectious diseases caused by host adaptation and the increasing number of invasive non-typhoidal *Salmonella* (iNTS) cases allude to the urgency of monitoring the evolution of *Salmonella* transmission[34,35]. Recent advancements in molecular biology and genomics, such as Whole genome sequencing (WGS) and transcriptomic analyses, have elucidated the complex regulatory networks that govern virulence gene expression and adaptive responses to environmental stresses[36–39]. In this research, we reported several outbreaks of pigeon paratyphoid in China, and conducted pathogen isolation and identification, genomic epidemiological investigations, and multiple phenotypic analyses of the outbreak events.

## Results

### Genomic epidemiology investigation of local pigeon Salmonellosis outbreaks

**Fig 1:**
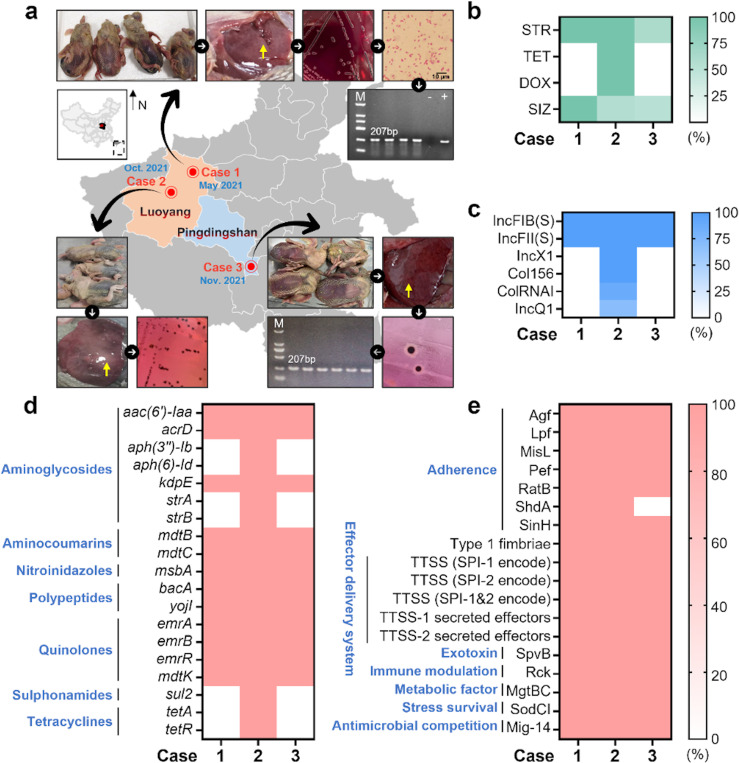
Pathogenetic analysis of three pigeon paratyphoid outbreaks in Henan Province. **A.** Temporal-spatial information at the time of outbreak and pathogen isolation and identification. In Case-1 and Case-2, the livers were enlarged with blunt edges, showing grey-white necrotic spots and numerous scattered grey-white lesions. In Case-3, the liver was congested and enlarged, with reddish-brown petechial hemorrhages on the surface and a fragile texture. A multi-step approach was adopted for the identification of pathogens, encompassing the sequential application of selective bacterial cultures, microscopic examination, and polymerase chain reaction (PCR). **B.** Results of antimicrobial susceptibility testing. Selected antimicrobials and adjudicated values refer to the CLSI M100 ED34 standard. Antimicrobial classes with 100% negative isolate test results will not be displayed. Escherichia coli ATCC 25922 and *Staphylococcus aureus* ATCC 25923, were used as control strains. **C.** Predicted results of plasmid carriage based on the Plasmidfinder database. ST19 isolates are considered potentially susceptible to aminoglycosides, sulfonamides, and tetracyclines. **D.** Predicted results of Antimicrobial Resistant Genes (ARGs) carriage based on the Resfinder database. **E.** Predicted results of Virulence Genes (VGs) carriage based on the VFDB database. Minimum DNA identity and minimum DNA coverage for genetic testing are both set to 80% for more comprehensive results. Unmatched genes will not be displayed. The base layer of the map is derived from the Standard Map Services (The review number is GS (2021) 5445; Links to images and terms of use are under the following domains: http://bzdt.ch.mnr.gov.cn/).

In 2021, three outbreaks of pigeon paratyphoid occurred in Henan Province, China: two in Luoyang City and one in Wugang City, under the jurisdiction of Pingdingshan City (Fig 1A). The affected squabs in all three outbreaks exhibited the “black belly” associated with pigeon-origin *S*. Typhimurium (poSTM) infection. Liver samples were collected for further bacterial determination. Four isolates were obtained from Case-1, 16 from Case-2, and 33 from Case-3. The biochemical tests confirmed them as belonging to the *Salmonella* genus (S1 Table). All Case-1 isolates and a proportion of Case-3 isolates (60.61%, 51.53%) were only resistant to streptomycin (STR) and sulfisoxazole (SIZ), but Case-2 isolates presented a more severe resistance profile, with 56.25% of strains exhibiting multi-drug resistance (MDR), showing resistance to aminoglycosides, tetracyclines, and sulfonamides (Fig 1B). PCR identification targeting the *invA* gene revealed that all 53 isolates carried this invasion-associated gene, as indicated by the amplification of the corresponding bands. Based on the results of various bacterial identification methods, it was concluded that the three outbreaks of pigeon paratyphoid in this study were caused by *Salmonella* infections.

We found that all isolates from each outbreak shared the exact identical sequence type (ST): ST128 in Case-1 and Case-3, and ST19 in Case-2. The serovar of all isolates from the three pigeon paratyphoid outbreaks was Typhimurium. The ST128 isolates from Case-1 and Case-3 carried the same plasmids, IncFIB(S) and IncFII(S) (Fig 1C). The ST19 isolates from Case-2 carried a more diverse array of plasmids. In addition to the plasmids found in ST128 isolates, the ST19 isolates also carried the IncX1 plasmid, which is widely reported to carry the Tn2 transposon harboring the *bla*_TEM-1B_ antibiotic resistance gene (ARG), the IncQ1 plasmid, which often carries the *tet(X4)* ARG, and the Col156 and ColRNAI plasmids, both of which play significant roles in the horizontal transfer of ARGs. The number of plasmid types was positively correlated with the amount of ARGs carried, indicating that ST19 isolates carried more ARGs than ST128 isolates (Fig 1D). The isolates from the three outbreaks were relatively consistent in their levels of virulence genes (VGs) (Fig 1E). However, isolates from Case-3 uniformly lacked the *shdA* gene, which is associated with intestinal adhesion and extracellular matrix binding[40]. The absence of this gene might lead to reduced virulence or adherence capabilities.

### Global population diversity of pigeon-origin *Salmonella*

**Fig 2:**
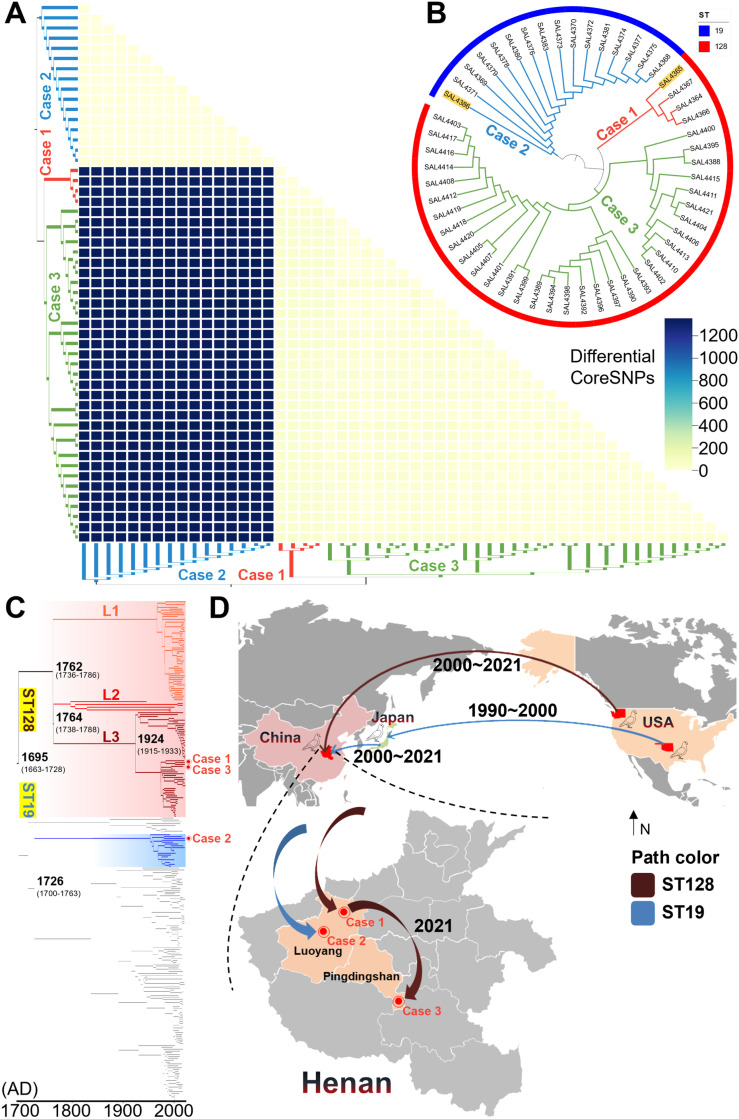
Genetic relationships and temporal-spatial tracing of outbreak isolates. **A.** Heat map of CoreSNPs differences. The number of differential coreSNPs increases with deeper color. The evolutionary trees on the left and below are consistent, and the colors are all relevant to the case. **B.** Phylogenetic relationships of the three outbreak isolates. The rooted tree was constructed from IQ-TREE v1.6.12 and visualized by iTOL v6. The reference is STM SL1344 and the outgroup is *Salmonella* Enteritidis ATCC13076, the outer circle indicates the sequence type. **C.** Time evolution tree of STM ST128 and ST19. The temporal evolutionary tree was constructed with Beast v2.7.6 and visualized with FigTree v1.4.4. The reference for the original phylogenetic tree is STM SL1344 and the outgroup is *Escherichia coli* K-12. Genetic branching of non-transparent backgrounds for pigeon host-adaptive STM phenotypes. The time ranges within the 95% HPD are indicated in parentheses. **D.** Simulation of the transmission pathways of *Salmonella* causing pigeon paratyphoid fever in Henan. A localized world map containing China, Japan, and the United States is shown at the top, and a map of Henan is shown at the bottom. Areas not associated with the spread of this outbreak pathogen are shown in gray. Transmission pathways and times were hypothesized based on meta-information from neighboring strains in a temporal evolutionary tree. The base layer of the map is derived from the Standard Map Services (The review number is GS (2021) 5445; Links to images and terms of use are under the following domains: http://bzdt.ch.mnr.gov.cn/).

The genetic distance among isolates was calculated by comparing the number of Differential Core SNPs (CoreSNPs). Referring to studies of *Salmonella* genetic evolution, two isolates with fewer than 100 Differential CoreSNPs can be considered the same strain[41,42]. The results showed that the pathogenic bacteria in Case-1 and Case-3 were the same, and Case-2 was also caused by a single strain (Fig 2A). After constructing a phylogenetic tree of the whole genomes of the 53 poSTM isolates, the research team selected the two isolates closest to the root for third-generation sequencing and subsequent experiments (Fig 2B). These were SAL4365, with genotype ST128, and SAL4386, with genotype ST19.

Previous research has shown that ST128 evolved from ST19[43]. Collecting all ST128 whole genomes and ST19 third-generation sequencing complete genomes from public databases, we constructed a divergence time evolutionary tree highly relevant to the outbreak isolates in this study. The regression of root-to-tip distances indicates the presence of a temporal structure within the dataset (β=3.22e-05, tMRCA>100), but the observed poor linearity between the different genealogical sequences suggests that they may be suitable for a loose molecular clock (Fig 2C). Bayesian typing tests showed that these three genetic branches are relatively independent. Therefore, this study named them L1, L2, and L3, with L3 being the branch to which the isolates from Case-1 and Case-3 in the three pigeon paratyphoid outbreaks in Henan belong.

Combining the evolutionary timeline and geographic information, the research team simulated the possible pathogen transmission routes for the three outbreaks (Fig 2D). The pathogens in Case-1 and Case-3 were initially introduced to China from meat pigeons imported from Washington State, USA. Within Henan Province, the bacteria spread via contaminated pigeon eggs, leading to successive outbreaks of pigeon paratyphoid caused by ST128 poSTM in Luoyang City and Wugang City. The pathogen in Case-2 was first introduced to Japan from Oklahoma, USA, before 2000, and then entered China after 2000, with meat pigeons again being the transmission host.

### Genomic signature for Pigeon-adaptive *Salmonella*

**Fig 3:**
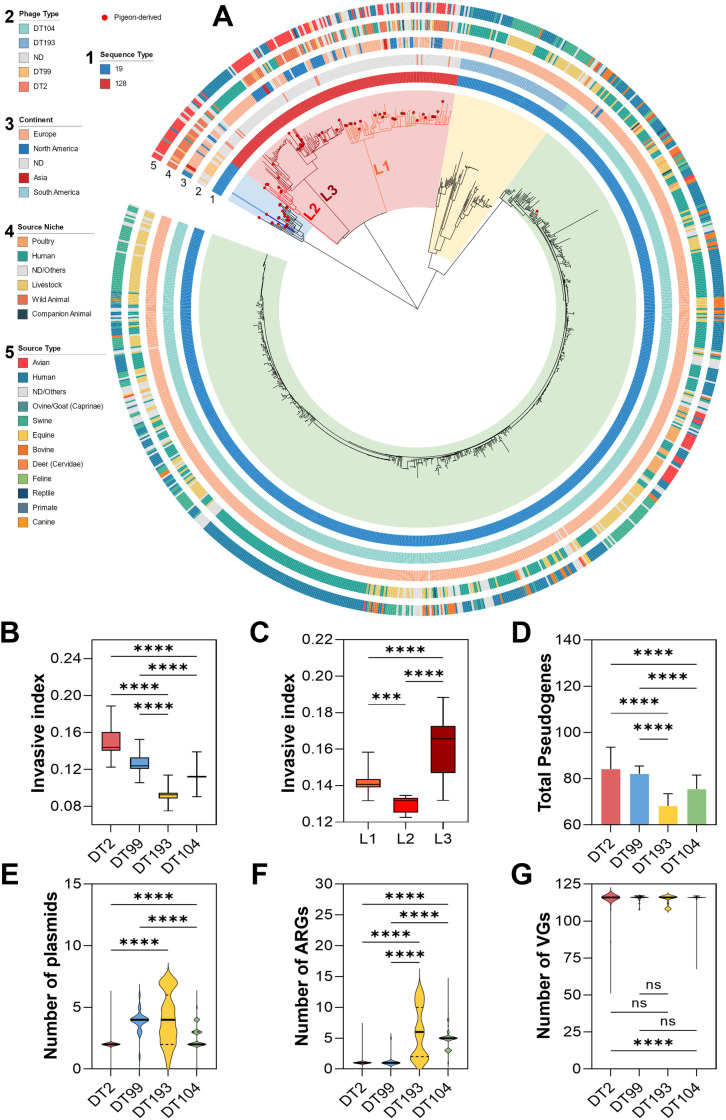
Comparative genomic analysis of STM with different host tropisms. **A.** Phylogenetic tree analysis dataset of four different phage types of STM, with DT2 in blue background, DT99 in red background, DT193 in yellow background, and DT104 in green background. A total of 1000 *Salmonella* WGS were included in the phylogenetic tree mapping. The reference is STM SL1344 and the outgroup is *Salmonella* Enteritidis ATCC13076. **B.** Differences in invasion indices, which predict the invasive capability of the strains, among different phage types. DT2 and DT99 are pigeon host-adaptive STM phage types. **C.** Differences in invasion indices among three lineages of STM ST128. Henan outbreak pathogen isolates are located in the L3 branch. **D.** Predicted results of pseudogene carriage based on the Pseudofinder v1.1.0. **E.** Predicted results of plasmid carriage based on the Plasmidfinder database. **F.** Predicted results of ARGs carriage based on the Resfinder database. **G.** Predicted results of VGs carriage based on the VFDB database.

Previous research indicates that STM phage types DT2 and DT99 exhibit host adaption for pigeons[44]. Consequently, we extensively collected genomes related to DT2 and DT99 strains, and compared them with available genomes of pan-host STM phage types DT193 and DT104 (Fig 3A). A multiple information system phylogenetic tree revealed that all three lineages of ST128 exclusively correspond to the DT2 phage type, indicating a specific relationship between ST128 and DT2. The isolates from Case-1 and Case-3 belong to the DT2 evolutionary branch. Additionally, the poSTM isolated in Case-2 falls within the DT99 evolutionary branch, suggesting that the poSTM isolates obtained in this study all exhibit pigeon host specificity. Compared to the pan-host STM DT104 and DT193, the intercontinental transmission of the two pigeon-adaptive phage types, DT2 and DT99, is more complex, frequently occurring in Europe, North America, and Asia. However, their host range is narrower, primarily isolated from pigeons and humans, and they are commonly found in the ecological niches of wild animals, humans, and poultry.

Invasion index analysis indicates that pigeon host-adaptive STM is more likely to invade the bloodstream, making it more prone to cause extraintestinal infections (Fig 3B). Among these, DT2 exhibits the highest invasion index, followed by DT99. The poSTM isolates from Case-1 and Case-3, which belong to the L3 lineage, show the highest invasion index and the most significant genetic distance from the root node in the phylogenetic tree (Fig 3C). Pseudogene analysis reveals that pigeon host-adaptive STM has the highest number of pseudogenes, with DT2 averaging 84.14 pseudogenes and DT99 averaging 82.08 (Fig 3D). This significant difference in the number of pseudogenes is attributed to the presence of a larger number of fragmented and too-short pseudogenes in pigeon host-adaptive STM, whereas differences in the number of too-long pseudogenes have a minimal impact on the overall pseudogene levels (S1A-C Fig).

It has been demonstrated by preceding epidemiological investigations that the majority of plasmids are disseminated via horizontal transfer between species[45,46]. Consequently, bacteria with a more extensive host spectrum are frequently associated with a greater number of plasmids and resistance genes. The number of ARGs carried by pigeon host-adaptive STM is significantly lower than that of pan-host STM (Fig 3F). DT2 carries an average of 1.08 types of ARGs, and DT99 carries an average of 1.17 types. The gene *aac(6’)-Iaa* appears in 99.5% of pigeon host-adaptive STM, but it is also widely present in pan-host STM. The lower detection level of ARGs in DT2 may be related to its fewer plasmid types, averaging 2.03 plasmids per isolate (Fig 3E). However, DT99 has a higher level of plasmid carriage, comparable to that of DT193. There is no significant difference in the levels of VGs among the four phage types, with the lowest VGs level in DT2 being only 1.91% less than the highest in DT104 (Fig 3G).

In addition to the above findings, the research team used third-generation sequencing data to analyze genes potentially associated with host adaptation changes, particularly those encoding structural proteins involved in virulence. The *fimH* gene of the ST128 poSTM isolates from Case-1 and Case-3 showed an unreported point mutation at the 48th base in the adhesin domain coding region (S1D Fig). This mutation results in a proline-to-serine substitution at a crucial mannose-binding site, which is highly likely to affect *Salmonella*’s affinity for polysaccharide structures on host cell surfaces. Further analysis of this mutation across all ST128 and pan-host ST19 STM sequences from public databases revealed that this point mutation is absent in pan-host ST19 but is present in more than 1% (specifically, 5.1%) of ST128 isolates (S1E-F Fig). This discovery of a new SNP is significant and warrants further research.

### *Salmonella* Typhimurium transition to host-adaptive tropism

To elucidate the variations in pigeon-adaptive *Salmonella*, we first carried out extensive phenotyping experiments. The *in vitro* experiments included survival and growth characteristics, motility, and biofilm formation analysis. The growth curves of the two poSTM strains were intermediate between those of pan-host and host-adaptive *Salmonella*, with a higher bacterial concentration during the stationary phase than the reference strains (S2A Fig). The growth curve of SAL4365 differed significantly from that of the reference strain, entering the logarithmic growth phase approximately 2 hours earlier and exhibiting faster growth at a high temperature of 42°C (S2B Fig). Motility tests indicated that aerobic conditions generally favored the swarming motility of STM. SAL4365 displayed poor motility, whereas SAL4386 showed the strongest motility, slightly surpassing that of the reference strain SL1344, with an average motility diameter of 8.83 mm under aerobic conditions (S2C Fig).

**Fig 4:**
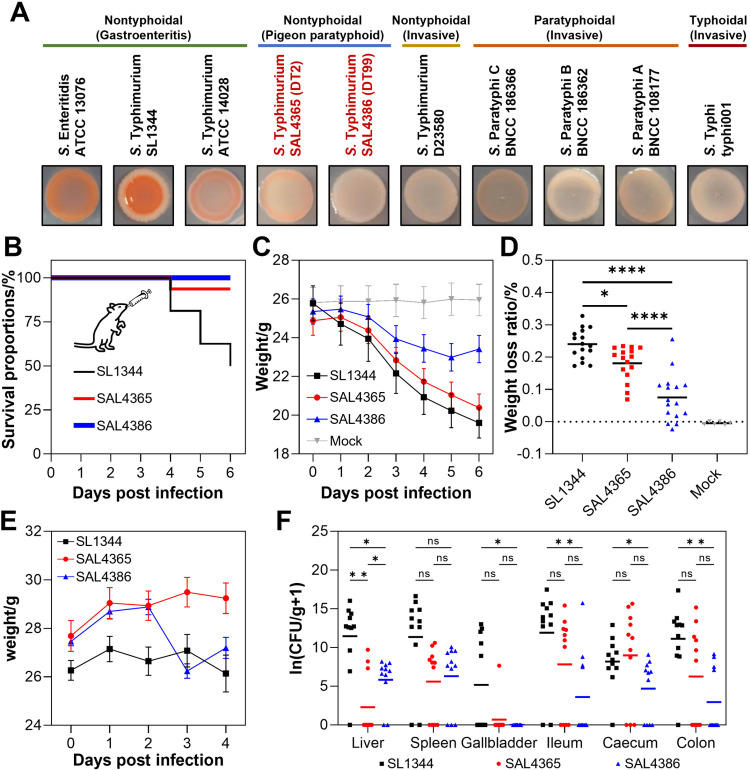
Alterations in pathogenicity and invasiveness of poSTM. **A.** Correspondence of rdar phenotype and invasiveness. The rdar phenotype tapers from left to right. Strains within the green horizontal line often cause gastroenteritis, while strains within the yellow, orange, and red horizontal lines often cause invasive symptoms. **B.** Survival curves, **C.** weight change curve, and **D.** weight loss rate of enteritis model mice. **E.** Weight change and **F.** organ bacterial load of typhoid model mice. SAL4365 represents ST128 (DT2) STM and SAL4386 represents ST19 (DT99) STM in b-f. The route of administration was by gavage in both cases.

Both poSTM strains exhibited colony morphologies on Congo red TA plates that were intermediate between those of invasive *Salmonella* and non-invasive rdar-positive phenotypes (Fig 4A). The colonies of SAL4365 had a flesh-colored outer ring, while the colonies of SAL4386 were white, smooth, and moist, closely resembling those of invasive *Salmonella*. Compared to the reference strain SL1344, which almost did not produce biofilms, both poSTM strains demonstrated significantly stronger biofilm formation capabilities (S2D Fig). Consistent with the reference strain, the biofilm formation ability of poSTM strains was enhanced under aerobic conditions. Notably, while SAL4386 had strong biofilm formation at 37°C, its ability was weaker than that of SAL4365 at 42°C under aerobic conditions (S2E Fig). Despite the increased temperature, the biofilm formation abilities of both poSTM strains did not significantly decline.

The *in vivo* experiments included investigations of pathogenicity and invasiveness using mouse models and virulence testing using chicken embryo models. In the murine enteritis model, mice began to die starting from the fourth day in the SL1344 and SAL4365 groups (Fig 4B). In the SL1344 group, mouse deaths continued in the following days, reaching a mortality rate of 50% by the sixth day. In contrast, the SAL4365 group saw no further deaths after the sixth day, with a final mortality rate of 6.25%. No deaths were observed in the SAL4386 group. Following STM infection, the SL1344 group experienced the fastest weight loss, followed by the SAL4365 group (Fig 4C). The SAL4386 group was the only one where the mice’s average weight began to recover on the day 6 post-infection. The Mock group’s mice did not experience continuous weight loss. Six days post-infection, the SL1344 group showed the highest average weight loss at 24.03%, significantly greater than that of the SAL4365 group (18.08%) and the SAL4386 group (7.53%) (Fig 4D). In the 16-day-old chicken embryo model, after allantoic cavity injection of STM, all chicken embryos, except those in the 10 CFU dose SAL4386 group, died within one day (S2F Fig). The remaining embryos in the SAL4386 group died on the second day. There were no significant differences between the groups, indicating that STM has a high virulence in chicken embryos.

The pathological examination of mice infected with the enteritis model revealed that the SL1344 group exhibited the most severe organ lesions overall, followed by the SAL4365 group, with the SAL4386 group showing the least severe lesions (S3 Fig). Notably, unlike the SL1344 group in which the colon failed to perform its desiccation function properly, the colons of mice in the SAL4365 and SAL4386 groups contained well-formed, unexpelled fecal pellets, indicating a relatively intact colonic function. In the SL1344 group, the liver showed the most significant pathological changes and severe damage, including lobular inflammation and fatty degeneration, with a few hepatocytes displaying small cytoplasmic vacuoles within the field of view (S4 Fig). In the two poSTM groups, although the liver structure was abnormal, the hepatic cords were orderly, and the sinusoids were uniformly sized, with only a slight infiltration of inflammatory cells. Corresponding with the gross appearance of splenomegaly, the SL1344 group also exhibited the most severe tissue damage in the spleen, with disorganized splenic nodules and indistinct red and white pulp boundaries. The tissue showed extensive neutrophil infiltration and a substantial deposition of hemosiderin. The spleen in the SAL4365 group also showed significant damage, with a small number of necrotic cells displaying nuclear fragmentation and pyknosis with deep staining. In contrast, the SAL4386 group had only minor splenic damage, characterized by neutrophil infiltration and a few multinucleated macrophages.

Comparative analysis of intestinal lesions across the three experimental groups revealed similar pathological features, primarily characterized by abnormal tissue structures, reduced crypt numbers, localized crypt loss, and minimal inflammatory cell infiltration. Among these, the villus height to crypt depth (VH/CD) ratio indicated the lowest value in the SL1344 group (1.22), suggesting the poorest intestinal mucosal integrity and digestive capacity, followed by the SAL4386 group (1.78) and the SAL4365 group (1.57) (S5A Fig). The SL1344 group had a significantly thicker cecal mucosa than the two poSTM groups (S5B Fig). Although the ileal lesions were most severe in the SL1344 group, both poSTM groups also displayed abnormal ileal structures, with epithelial cell ulceration and shedding in certain mucosal regions exposing the lamina propria. In contrast to the cecum, the SL1344 group exhibited relatively more severe colonic damage, with localized mucosal epithelial cell shedding and exposed lamina propria, resulting in a markedly lower compensatory mucosal thickening (173.07 μm) compared to the SAL4365 group (266.70 μm) and the SAL4386 group (379.15 μm) (S5C Fig).

The results of the murine typhoid model experiment demonstrated that, in the presence of normal gut microbiota, only the SAL4386 group exhibited a significant weight loss by the third day, whereas the SAL4365 group showed a trend of weight gain (Fig 4E). Except for the cecum, the bacterial load in other organs of the SL1344 group was higher compared to the poSTM experimental groups (Fig 4F). Interestingly, although the bacterial load in the ileum, colon, and cecum was higher in the SAL4365 group than in the SAL4386 group, this pattern was reversed in the liver and spleen, which are critical immune organs.

### Pigeon outbreak isolates show higher virulence and pathogen burden in cogent models

**Fig 5:**
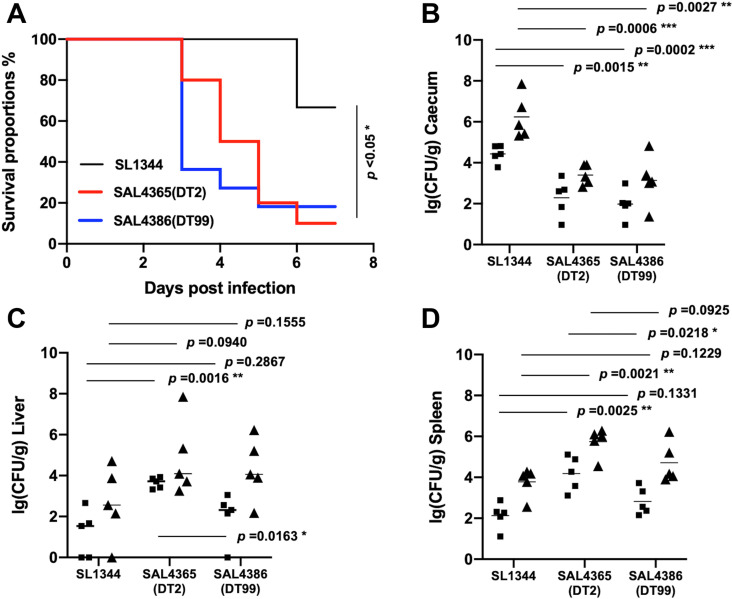
Pathoadaptation of poSTM in pigeon models. **A.** The survival curve for three examined *Salmonella* isolates. To examine the virulence potential, 1x10^9^ CFU pathogens were orally inoculated into the day-old pigeons. Each group (n=10) was challenged with an individual pathogen. *Salmonella* Typhimurium (STM) lab strain SL1344 is the control, SAL4365 represents ST128 phage type 2 or DT2, and SAL4386 ST19 phage type 99 or DT99. To investigate the pathogen load in the tissues, a lower challenged dose 5x10^7^ CFU was used for the day-old pigeon. Bacterial burden in the Caecum **B.**, Liver **C.** and Spleen **D.** of the pigeons were shown. Among the three challenged groups (n=10), five pigeons were examined on day 3 post-infection (black square), and the remaining five pigeons were studied on day 7 post-infection (black up triangle). The y axis denotes log10 CFU of bacterial pathogen/gram. Each dot represents triplicate results for one pigeon. The statistics were conducted by an unpaired t-test, with the *p*-value indicated accordingly.

To fulfil Koch’s postulates for the pigeon outbreak isolates, we recruited the *Salmonella*-free day-old pigeons to examine the virulence of the bacterial isolates in a higher dose (1 x 10^9 CFU), with SL1344 as a control. We found that both DT2 (SAL4365), a DT2-L3 isolate in this study, and DT99 (SAL4365) isolates had a fast-killing rate, and they showed a statistically significant to the control strain SL1344 (*p*<0.05) (Fig 5A). To further investigate the pathogen burden in the examined tissues, we used a low dose (5 x 10^7) to ensure a higher tolerance in young pigeons during the 7-day infection assay. To measure the pathogen loads in the caecum (Fig 5B), liver (Fig 5C) and spleen (Fig 5D). Both DT2 and DT99 isolates had statistically low pathogen load in the caecum compared to the control strains on day 3 and day 7 post-infection. For systemic tissues, only the isolate shows a higher bacterial load in the liver when compared with SL1344. Of note, the DT2 isolate had a higher bacterial load than the DT99 isolate, indicating a more pigeon-adaptive feature in DT2-L3 isolate. In support of this pigeon-adaptive trend, in general, the bacterial load is higher in DT2-L3 isolate than the DT99 isolate, and the DT99 isolate is higher than the SL1344, which is considered a host-inclusive strain with a general host-tropism as a control in this study. We concluded that both isolates from DT2-L3 and DT99 exhibited a pigeon-adaptive pathway in the cogent models.

## Discussion

Pigeon paratyphoid is a common bacterial infectious disease in pigeon lofts, characterized by symptoms such as diarrhea, arthritis, and neurological symptoms, which can lead to high mortality rates in squabs, posing a severe threat[47]. Not only do infected pigeons require prolonged treatment, but even after recovery, they remain carriers, continuing to shed and spread the pathogen. Pigeon paratyphoid often occurs concurrently with pigeon plague, colibacillosis, and trichomoniasis, resulting in more severe economic losses. Apart from the widespread transmission of the pathogen and its high virulence in pigeons, the unique breeding mode is also a crucial factor contributing to the high incidence of pigeon paratyphoid[48]. Squabs must be incubated and nurtured by paired adult pigeons during the developmental stage when they cannot feed or drink independently. This breeding practice leads to frequent contact among susceptible birds, making an “all-in-all-out” system impossible, providing favorable conditions for spreading *Salmonella* within the loft, and increasing the difficulty of disinfection and disease control[49–51].

This study conducted on-site sampling, pathogen isolation and identification, and whole-genome sequencing (WGS) analysis of three pigeon paratyphoid outbreaks in Henan Province. The causative agents were identified as STM of the genotype ST128 in Case-1 and Case-3, and STM of the genotype ST19 in Case-2. These two genotypes belong to distinct evolutionary branches (DT2 and DT99) that exhibit host adaption for pigeons[52]. Antimicrobial susceptibility testing revealed that ST128 poSTM was resistant to fewer drugs, while ST19 poSTM exhibited multidrug resistance (MDR). Although the Case-2 isolates had a more complex resistance profile, the Case-1 and Case-3 isolates showed higher resistance to sulfisoxazole. In the aftermath of the outbreaks, pigeon lofts should avoid using antibiotics such as tetracyclines and take measures to prevent the further spread of MDR isolates. The analysis of associated ARGs and plasmid carriage was consistent with the observed resistance phenotypes. The research team hypothesized that the limited host range of ST128 might have reduced opportunities for acquiring mobile genetic elements from other bacteria[45]. While the spatiotemporal evolutionary analysis explained the origins of the poSTM transmission in Henan, the analysis could be improved by incorporating more genome sequences due to the limited number of relevant strains available in public databases.

We employed comparative genomic and phenotypic approaches to investigate the differences between pigeon outbreak STM isolates from this study and ubiquitous STM strains, aiming to provide insights into the mechanisms of bacterial host adaptation[53]. Pigeon-adaptive STM exhibited significantly higher levels of pseudogenes, potentially reducing immunogenicity and facilitating adaptation to the pigeon host environment[54]. Additionally, higher invasion indices, enhanced biofilm formation, and the rdar-negative phenotype suggest that poSTM may have undergone changes to enhance its invasiveness, consistent with the evasion of host innate immunity during adaptation[55]. Although the bacterial burden experiment using the murine typhoid model did not support the hypothesis of increased invasiveness, it strongly demonstrated that STM’s adaptation to pigeons coincided with a reduced ability to invade other animal hosts. This host-adaptive preference was more pronounced in the infection experiments with host-restricted *Salmonella* serovars. Consequently, mice and chicken embryos are not ideal experimental models for studying the host adaptation mechanisms of pigeon-adaptive STM, and pigeons without specific pathogens should be used instead. Furthermore, at high temperatures (42°C), poSTM (especially ST128) exhibited higher growth rates and lower biofilm formation reduction, better adapting to the higher body temperature of birds and promoting persistent infections. Additionally, the cogent models demonstrated both ST-128 DT2 L3 and ST19-DT99 isolates exhibited a high killing and pathogen burden in the systemic tissues.

While this study did not explore the mechanisms underlying STM’s adaptation to pigeons, the genomic epidemiological investigation and phenotypic assays provide a theoretical basis for the treatment and prevention of local pigeon paratyphoid outbreaks[56]. Breeding enterprises should enhance management practices, regularly disinfect the environment and, hatch eggs, and prevent the entry of wild birds such as sparrows and doves into pigeon lofts[57]. Additionally, during pigeon paratyphoid outbreaks, prompt isolation of infected pigeons, disinfection of the loft, and alternating or combined antibiotic treatments should be implemented to avoid the emergence of resistant strains.

## Materials and methods

### Ethics Statement

All the pigeons in field investigations and infection assays were approved and conducted at the College of Veterinary Medicine, Henan University of Animal Husbandry and Economy. The murine and chicken embryo assays were approved by the Zhejiang University ethical review process.

### Case Information

In 2021, there were three outbreaks of pigeon paratyphoid in Henan Province. The background is as follows: Case-1: The first outbreak occurred in early May at Farm A, located in the suburbs of Luoyang City. This farm primarily sells hatching eggs, resulting in a relatively low breeding density, with most pigeons in cages. The outbreak led to the death of only 14 squabs, each approximately 3 days old. Pigeons at this breeding farm are generally kept for conservation purposes and are not treated with antibiotics for prevention. Instead, a traditional Chinese medicine premix containing Astragalus polysaccharide is used for health maintenance.

Case-2: The second outbreak occurred in mid-October at Farm B in Yiyang County, Luoyang City, resulting in the death of over 200 squabs aged approximately 2 to 3 days. This farm specializes in meat pigeons and houses about 100,000 pairs for meat and squab purposes. Due to poor pen conditions and the presence of many wild birds, such as sparrows, the risk of disease transmission is high. Therefore, the farm management supplements routine health maintenance with Astragalus polysaccharide by administering an antibiotic premix every four days for infectious disease prevention, primarily using enrofloxacin, ciprofloxacin, and neomycin.

Case-3: The third outbreak began on November 5th at Company C in Wugang City and lasted nearly 15 days. The outbreak resulted in the death of 97 squabs, each approximately 7 days old, in four breeding houses and led to a high embryonic mortality rate of 11% in that batch of hatching eggs. Company C is a large enterprise, housing 300,000 pairs of core breeding pigeons across 13 meat pigeon breeds, and operates two major breeding bases. Despite the high level of automation in their management, this outbreak might have been caused by asymptomatic carrier status in newly paired adult pigeons nursing their first batch of squabs. After the outbreak, Company C promptly added enrofloxacin to the drinking water for preventive treatment, reducing the daily squab mortality to six after four days of medication.

### Isolation and identification of *Salmonella* Typhimurium

Pathological dissection was performed on deceased pigeons, and liver tissue was collected for *Salmonella* isolation[58]. The liver tissue was homogenized in PBS buffer using a tissue oscillator. A sterile inoculation loop was used to streak the liver homogenate onto XLD agar plates, which were incubated overnight at 37°C. After repeated streaking for purification, suspicious colonies were inoculated into LB broth and cultured at 37°C with shaking at 180 rpm for 8 to 12 hours. Subsequently, 500 μL of the LB culture was mixed with 500 μL of 40% glycerol in cryovials, labeled with strain information, and stored at -80°C. Following national standards for *Salmonella* detection, such as “PCR Detection Method for *Salmonella* in Chicken Intestines (GB/T 40049-2021)” and “*Salmonella* Detection Method in Experimental Animals (GB/T 14926.1-2001)”, frozen bacterial stocks were streaked onto XLD agar plates using an inoculation loop and incubated overnight at 37°C. The following identification tests were conducted sequentially: Gram staining microscopy, biochemical identification, and PCR identification using primers designed for the invasion-related gene *invA*.

### Antimicrobial susceptibility testing

This study references the guidelines from the Clinical and Laboratory Standards Institute (CLSI) for antimicrobial susceptibility testing of *Salmonella* as outlined in M100 ED34[59–61]. The susceptibility of the test strains to antimicrobial agents was determined using the disk diffusion method (M02 ED14) on Mueller-Hinton Agar (MHA) plates. Standard strains, Escherichia coli ATCC 25922 and Staphylococcus aureus ATCC 25923 were used as control strains. Since the isolates from the same outbreak event are clonal, repeated tests on single isolates were not conducted for the susceptibility tests. The experimental procedure follows: Similar pure culture colonies were picked from LB agar plates and inoculated into Mueller-Hinton Broth (MHB), then shaken at 37°C for 6 to 8 hours. The culture was diluted with sterile MHB to a turbidity of 0.5 McFarland units (approximately 1 × 10^8 CFU/mL). A sterile cotton swab was dipped into the bacterial suspension, and excess liquid was removed by pressing against the tube wall. The swab was then streaked evenly across the entire surface of an MHA plate, ensuring a uniform lawn of bacteria, and left to stand at room temperature for 3 to 5 minutes in a sterile environment. Antimicrobial disks were placed onto the agar surface using a disk dispenser or sterile tweezers, pressing lightly with the tip of the tweezers to ensure complete contact. Each disk was spaced at least 24 mm apart, with the center of each disk at least 15 mm from the edge of the plate. A maximum of six disks were placed on each 90 mm diameter plate. The types of antimicrobial agents used on the disks are listed (S2 Table).

### Whole genome sequencing and assembly

This study utilized both second-generation and third-generation sequencing technologies to obtain the whole genome data of STM outbreak isolates[62–64]. The specific process involved genome extraction, whole genome sequencing (WGS), and processing and assembly of sequencing data. The bacterial genomic DNA was extracted using a bacterial genomic DNA extraction kit purchased from Nanjing Novozymes Biotechnology Co., Ltd. The concentration and purity of the collected DNA products were measured with a Nanodrop micro-spectrophotometer from Thermo Fisher Scientific, and the DNA was stored at -20°C to prevent degradation. DNA samples from 53 isolates that met quality requirements were subjected to WGS using the Illumina NovaSeq 6000 platform. The raw sequencing data underwent md5 checksum verification and quality check using FastQC v0.12.1[65]. The Centrifuge integrated into the Snakemake v8.10.4 pipeline was used to analyze microbiological contamination in the quality-checked *.fastq files with a pass threshold of 0.8[66,67]. Data that failed quality control or showed severe contamination were discarded, and new genomic DNA was extracted and sequenced. Adapter sequences and low-quality reads were removed using Trimmomatic v0.39. The high-quality *.fasta files were then assembled de novo using SPAdes v3.15.5, which was nested in the Galaxy server[68,69]. Assembled WGS scaffolds were disposed to the NCBI BioProject database under accession number PRJNA854762 after removing the sequence less than 200 bp in length.

The complete bacterial genome sequence of strains SAL4365 and SAL4386 was obtained Nanopore Technology (Beijing Novogene Co. Ltd.) [70]. Additionally, this approach enabled the acquisition of plasmid genome information. Similar to the second-generation sequencing data processing, the PacBio Sequel platform was used for third-generation sequencing. The process involved constructing a 10 kb single-molecule real-time sequencing library, sequencing, and filtering out low-quality reads (less than 500 bp). The third-generation sequencing data were assembled into contigs and validated using SMRT Link v5.0.1.

### Typing prediction and comparative genomics analysis

The assembled whole genome sequences were used for various bioinformatics analyses. First, KmerFinder v3.2 was used to validate the species classification of the isolates[71]. The sequence type and serotype of *Salmonella* were identified using MLST v2.22.0 and SeqSero2 v1.2.1, respectively[72,73]. Additionally, the isolates obtained in this study, along with publicly available STM whole genome sequences, were analyzed using ABRicate v1.0.1, which integrates multiple bioinformatics tools. These included ResFinder v4.5.0 for predicting antimicrobial resistance genes (ARGs), PlasmidFinder v2.1 for predicting plasmids, and the VFDB database for VGs[74–76]. Moreover, the research team applied a well-established random forest model to the whole genome sequences to predict the invasive capability of the strains by calculating the invasion index[77]. To explore the correlation between host adaptability of poSTM and the accumulation of pseudogenes, Prokka v1.14.6 was used for gene annotation[78]. The MakeBlastDB v0.1.0 tool was then used to create a database from non-redundant protein sequences downloaded from UniProtKB[79]. Finally, Pseudofinder v1.1.0 was employed to compare and quantify the number of pseudogenes reported to be associated with host adaptation or generalism in STM[80].

### Phylogenetic analysis

This study first conducted phylogenetic analysis on the whole genome sequences of 53 STM isolates obtained from the experiment using second-generation sequencing. The clonal strains closest to the root node of the phylogenetic tree were selected for third-generation sequencing. These sequences were then compared with all whole genome sequences of the same genotype from public databases (EnteroBase and GenBank) to construct a comprehensive phylogenetic tree[81]. Additionally, a temporal and spatial evolutionary tree was built using complete sequences of STM ST19 from public databases to trace the international transmission routes of the Henan avian paratyphoid outbreak pathogen. The whole genome sequences listed in S3 Table met the following quality criteria and inclusion standards: file size between 4 and 6 MB, fewer than 500 contigs, N50 value of 30,000 or higher, and GC content between 50% and 56%. Using the whole genome sequence of the *Salmonella* reference strain SL1344 as the reference genome, Snippy v4.6.0 was utilized to detect core genome single nucleotide polymorphism sites (CoreSNPs)[82]. For Bayesian typing of global whole genome data of ST128 STM, the R package FastBAPS was employed[83]. Pan-genome analysis was performed using Roary v3.13.0 on the *.gff files annotated by Prokka v1.14.6[84]. The SNP alignment generated by Snippy was used to construct a phylogenetic tree with the IQ-TREE v1.6.12 software, applying the Generalized Time-reversible (GTR) model based on the maximum likelihood (ML) method[85]. The resulting *.treefile was then visualized using iTOL v6[86]. The SNP distance matrix transformed from SNP alignment is used to plot the SNP genetic distances heatmap.

The temporal evolutionary analysis builds on the standard phylogenetic analysis and involves the following steps: Treetime is used to assess the time signal in the *.treefile generated by IQ-TREE and the unbiased *.aln alignment file reconstructed by Gubbins v3.4.4, along with temporal information[87,88]. Genomes with abnormal signals are removed. Beast v2.7.6 is used to construct a temporal evolutionary tree based on the NEX format prior tree, predicting divergence times[89]. BEAST analyses were conducted on concatenated cgSNP alignments employing GTR+Γ substitution model, Bayesian skyline models, and relaxed lognormal molecular clocks[90]. The chain length was set to 100 million, with sampling occurring every 10,000 iterations. Tracer v1.7.2 is employed to check for convergence in the divergence time predictions in the *.log files, ensuring that the Effective Sample Size (ESS) parameters for each tree exceed 200 before merging[91]. TreeAnnotator software is used to merge the tree sets after discarding the initial 20%, resulting in the final temporal evolutionary tree[92]. FigTree v1.4.4 is used to visually edit and present the merged *.tree file.

### Growth characteristics assay

The test strains were resuscitated by streaking onto plates, and single colonies were picked using a sterile inoculation loop into 1 mL of LB broth. These cultures were incubated overnight at 37°C with shaking at 180 rpm. The following day, fresh cultures were inoculated into sterile LB broth at a 1:100 ratio and incubated at 37°C with shaking at 180 rpm. Optical density at 620 nm (OD_620_) was measured every hour for 12 hours. The growth curves were plotted and fitted using the Logistic growth model to compare the growth dynamics of different strains. To investigate the potential impact of temperature on bacterial growth, considering that pigeons have a higher body temperature than mammals, an additional experimental group was set up at 42°C with all other conditions being the same. The differences in growth curves between 37°C and 42°C were compared. The normality test was performed using the Kolmogorov-Smirnov test and a two-way ANOVA was carried out. The generalist STM control strain (SL1344) and the host-adaptive *Salmonella* Gallinarum strain (R51) used as controls were maintained in our laboratory.

### Mobility testing

The primary “organ” for *Salmonella* motility is the flagellum, which, along with chemotaxis, facilitates bacterial penetration into the intestinal mucus layer, constituting an essential component of invasiveness. This study tested the swarming motility of the strains under aerobic and anaerobic conditions using three laboratory-preserved STM reference strains: SL1344, ATCC 14028s, and D23580. Inoculate the test strains into antibiotic-free LB broth and incubate at 37°C with shaking at 180 rpm until the OD_620_ reaches 0.06-0.08. Add 3 μL of the bacterial culture onto the surface of LB semi-solid agar plates and allow the droplets to air dry in a sterile environment. Perform six replicates for each test strain and incubate the plates in a 37°C incubator for 6 hours. Measure the widest diameter of bacterial spread using a vernier calliper and record the results.

### Rdar phenotype testing

The rdar (red, dry, and rough) phenotype is considered a relatively conserved biofilm morphology of non-typhoidal *Salmonella* (NTS), formed through interactions among fimbriae, cellulose, and other polysaccharides[93]. This study aimed to investigate the invasive capabilities of the test strains using the rdar phenotype assay. Reference strains included ATCC 13076, ATCC 14028, BNCC 186366, BNCC 186362, BNCC 108177, SL1344, D23580, and typhi001, all purchased and preserved by the laboratory. Prepare Tryptone agar (TA) by adding 40-60 mg of Congo red indicator per liter of TA before autoclaving to achieve high-pressure sterilization. Streak the test strains on TA plates and pick single colonies for inoculation into 5 mL of LB broth for overnight culture. Enrich the overnight culture until the OD_620_ reaches 1.0, indicating a dense bacterial suspension. Spot 10 μL of the enriched culture onto the Congo red TA plates and incubate them at 25°C overnight. The following day, observe the colony morphology and characteristics. Optionally, inoculate 1 × 10^9 CFU of the test strains into 100 mL of 1% Tryptone broth (pH 7.4) and incubate them at 28°C with shaking at 200 rpm for 24-48 hours. Monitor the growth in the liquid medium during this period.

### Biofilm formation testing

To quantitatively assess biofilm formation using crystal violet staining, bacterial biofilms can bind with crystal violet dye, and this binding can be quantified by the absorbance values measured using a spectrophotometer. The specific experimental steps are as follows: Revive the test strains by streaking them onto agar plates. Inoculate single colonies with a sterile inoculation loop into 1 mL of TSB and incubate at 37°C with shaking at 180 rpm for 12-16 hours. Dilute the fresh bacterial culture from overnight incubation in TSB to achieve an OD_620_ of approximately 0.08. Further dilute this culture 10-fold in TSB, vortex thoroughly, and inoculate 200 μL into each well of a 96-well plate. Set up the 96-well plate under anaerobic and aerobic conditions, with each bacterial strain having 6 parallel wells in each environment. Negative control wells contain sterile TSB, and positive control wells contain Escherichia coli ATCC 25922. Surround each well with 200-300 μL of ddH2O to prevent contamination and edge effects. Incubate the plate in a 37°C constant temperature chamber and allow static incubation for 48 hours. After 48 hours, remove the liquid from each well, wash gently three times with ddH2O to remove loosely bound bacteria, and air-dry the plate in a 60°C oven for 30 minutes. Stain each well with 200 μL of 0.4% crystal violet at room temperature for 25 minutes. Remove excess stain by rinsing each well with ddH2O until no further color washes out, and air-dry to remove residual liquid. Treat each well with 200 μL of 75% ethanol (ethanol and acetone in a 3:1 ratio) at room temperature with gentle shaking for 20 minutes to solubilize and decolorize the dye within the wells. Measure the absorbance values at 550 nm wavelength using a spectrophotometer to quantify the amount of crystal violet dye bound, reflecting the biofilm formation capability of the bacterial strains.

### Chicken embryo infection assay

Due to the restricted availability of SPF pigeons, previous validation trials on pigeon host adaptation have predominantly employed SPF chicken embryos or chicks as animal models[32]. The reference strain is SL1344. SPF chicken embryos were purchased from Ningbo Purebred Agricultural Technology Co., Ltd. Three to four days before use, fumigation disinfection with formaldehyde was conducted at a rate of 5 g potassium permanganate, 10 mL formaldehyde, and 10 mL distilled water per cubic meter. Residual disinfectants were removed 0.5 days in advance with proper ventilation. SPF chicken embryos were maintained in a constant temperature incubator at 37.5°C and 50% to 60% humidity until 16 days of age. Embryo viability was confirmed using a candling device before inoculation, with non-viable embryos discarded. From days 14 to 15 of incubation, bacterial strains were revived by streaking and incubated overnight at 37°C and 180 rpm. On day 16 of incubation, bacterial suspensions were washed with PBS (pH 7.2), diluted to an OD_620_ of approximately 0.1 (corresponding to a bacterial concentration of approximately 1 × 10^8 CFU/mL), further diluted to 10^2, 10^3, and 10^4 CFU/mL, and then injected into the amniotic sac at 100 μL per embryo, with 16 embryos per group. Daily candling was performed to record mortality rates.

### Pathogenicity testing of murine enteritis model

The reference strain used was SL1344. 129S2/SvPasCrl mice were purchased from Beijing Vital River Laboratory Animal Technology Co., Ltd. One week before infection, 7- to 11-week-old 129S2/SvPasCrl mice were housed in disinfected cages to acclimatize and avoid stress (S5D Fig). Mice were grouped and toe-tagged, with 16 mice per group housed in two cages. A 200 mg/mL solution of streptomycin sulfate in PBS (pH 7.2) was prepared, filter-sterilized, and administered via gavage at a dose of 100 μL per mouse using an alcohol-disinfected gavage needle 24 hours before the challenge. Before inoculation, the bacterial strains were revived by streaking on LB agar, followed by overnight culture. After centrifugation, the culture medium was removed, and the bacteria were resuspended in PBS. The suspension was adjusted to an OD_620_ of approximately 0.1 (corresponding to a bacterial concentration of approximately 1 × 10^8 CFU/mL) and used for inoculation. Three to four hours before inoculation, mice were removed from their cages and orally gavaged with 200 μL of the bacterial suspension per mouse. The control group received an equal volume of sterile PBS buffer. After gavage, mice were observed for 15 minutes to prevent accidental death due to operational errors before being returned to their cages. Body weight and mortality monitoring continued daily from the onset of antibiotic treatment until the 6th-day post-infection. The weight of deceased mice was included in subsequent calculations of average weight. On the 6th day post-infection, three mice from each group were selected for necropsy. Besides examining organ tissues for pathological changes, paraffin sections of the liver, spleen, ileum, cecum, and colon were prepared and stained with hematoxylin and eosin (H&E) for histopathological evaluation. In the ileum sections, measurements of villus length and crypt depth were taken from five intact villi. Mucosal layer thickness in the cecum or colon sections was also measured at five locations and subjected to statistical analysis.

### Invasion testing of murine typhoid model

This experiment utilized a murine typhoid model to investigate the invasive ability of the test strain (S5E Fig). Unlike the enteritis model, no pre-treatment with antibiotics was administered to the mice. The preparation before inoculation, reference strain, and experimental mice were consistent with the enteritis pathogenicity test, with each group consisting of 11 mice. Bacteria were orally gavaged at a dose of 100 μL per mouse (approximately 1 × 10^7 CFU per mouse), while the control group received an equal volume of sterile PBS buffer. The body weight and health status of the mice were monitored continuously until the 4th day post-infection. On day 4 post-infection, mice were euthanized by cervical dislocation under sterile conditions, followed by aseptic dissection. The liver, spleen, gallbladder, ileum, cecum, and colon tissues were immersed in PBS buffer. Portions of the tissues were weighed, homogenized in a 0.2% TritonX-100 solution with grinding beads, and subsequently diluted. Ten microliters of the dilutions were plated on SS agar plates and incubated at 37°C for 8 to 12 hours. Colonies were then counted and analyzed graphically.

### Pigeon infection assays

For infections, cryopreserved *Salmonella* pigeon isolates (SAL4365, an ST128-DT2 L3 isolate; SAL4386, an ST19-DT99 isolate) and control strain SL1344 were defrosted rapidly in a warm water bath and diluted to 10^9 and 5 x 10^7 CFU in phosphate-buffered saline (PBS). To examine the virulent potentials, we used a higher dose (10^9 CFU) to orally inoculate the day-old pigeons for three groups (n=10 for each group). We only examine the death outcome during the 7-day infection course. To further examine the pathogen burden, the day-old bird was used to orally gavage with a lower dose (5 x 10^7 CFU); at 3- and 7-day post-challenge, five birds from each three groups, the caecum, liver and spleen samples were collected to examine the bacterial load. One-day-old pigeons (Columba livia domestica) of mixed sex were obtained from a commercial hatchery. Sixty birds were randomly divided into six groups for the above two experimental infections. Before experimental procedure, all birds were confirmed as *Salmonella*-free by cloacal swabs and fecal samplings, which were streaked onto selective XLT4 (Luqiao Biotech., Beijing, China) and grown for 24 h at 37 °C.

### Statistical analysis

GraphPad Prism 9.5.1 was utilized for statistical analysis and visualization of data unrelated to genomic sequences. Results in bar graphs are presented as mean ± standard deviation (mean ± SD), while in line graphs, results are shown as mean ± standard error of the mean (mean ± SEM). Statistical analyses were conducted using t-tests, one-way analysis of variance (ANOVA), and Two-way ANOVA. Significant differences between groups were indicated using symbols: “✱” for *p* < 0.05, indicating significance; “✱✱” for *p* < 0.01, “✱✱✱” for *p* < 0.001, or “✱✱✱✱” for *p* < 0.0001, indicating highly significant differences. “ns” denotes no significant difference.

## Supporting information

S1 Fig Analysis of pseudogenes and mutations in pilus genes. A. Predicted results of fragmented pseudogenes carriage. **B.** Predicted results of too-short pseudogenes carriage. **C.** Predicted results of too-long pseudogenes carriage. **D.** Schematic diagram of *fimH* mutation bases and the amino acid spatial locations where point mutations affect translation. **E.** Mutation rate in the STM ST19 population. **F.** Mutation rate in the STM ST128 population.
(PDF)

S2 Fig In vitro phenotypic testing and chicken embryo infection results. A. Growth curve at 37°C under aerobic conditions. **B.** OD_620_ ratio changes in aerobic cultures at different temperatures. **C.** Motility diameter under different oxygen conditions. **D.** Biofilm formation capability at 37°C. **E.** Biofilm formation capability at 42°C. **F.** Chicken embryo survival curve.
(PDF)

S3 Fig Pathological anatomy of enteritis model mice. The red dashed lines connect the junction of the ileum and cecum in each group. In the SL1344 group, the liver presented with multiple pale lesions, a friable texture, and rounded edges. While hepatomegaly was also observed in the two poSTM experimental groups, the liver’s appearance in terms of color was similar to that of the Mock group, indicating minimal changes. The gallbladder and spleen in the SAL4365 and SAL4386 groups showed pathological enlargement, with sizes intermediate between those of the SL1344 and Mock groups. The colon of the SL1344 group mice was notably shorter than that of the poSTM experimental groups and exhibited numerous varicella-like nodules, with both the SL1344 and SAL4365 groups presenting with petechial hemorrhages.
(PDF)

S4 Fig Histopathological imaging of enteritis model mice tissue stained with HE (200×). The organs from the Mock group exhibited normal structures under the microscope, with clear cellular arrangements and no signs of edema or inflammatory cell infiltration. In liver sections, red arrows indicate vacuoles within hepatocytes, yellow arrows indicate inflammatory cell infiltration, and black arrows indicate hepatocyte necrosis; in spleen sections, yellow arrows indicate neutrophil infiltration, green arrows indicate iron deposition, blue arrows indicate an increased number of multinucleated macrophages, and red arrows indicate splenic cell necrosis. In intestinal sections, blue arrows indicate localized crypt structure loss, yellow arrows indicate inflammatory cell infiltration, black arrows indicate cell necrosis, and red arrows indicate localized mucosal epithelial cell ulceration and exposure of the lamina propria.
(PDF)

S5 Fig Intestinal lesions of enteritis model mice and animal experimental design. A. Ratio of villus height to crypt depth in the ileum. **B.** The mucosal layer thickness of the cecum. **C.** The mucosal layer thickness of the colon. **D.** Schedule of pathogenicity trials in enteritis model mice. **E.** Schedule of invasion trials in typhoid model mice.
(PDF)

S1 Table Biochemical Identification Result. (XLSX)

S2 Table Antimicrobial Susceptibility Testing Result. (XLSX)

S3 Table *Salmonella* Typhimurium Whole Genome from Public Database. (XLSX)
