# Supplementary material for: A genomic and phenotypic investigation of pigeon-adaptive Salmonella
Source: PLoS Pathog. 2025 Mar 17;21(3):e1012992. doi: 10.1371/journal.ppat.1012992 (PMC11957392; doi:10.1371/journal.ppat.1012992)

S1 Fig. Analysis of pseudogenes and mutations in pilus genes. A. Predicted results of fragmented pseudogenes carriage. **B.** Predicted results of too-short pseudogenes carriage. **C.** Predicted results of too-long pseudogenes carriage. **D.** Schematic diagram of *fimH* mutation bases and the amino acid spatial locations where point mutations affect translation. **E.** Mutation rate in the STM ST19 population. **F.** Mutation rate in the STM ST128 population.

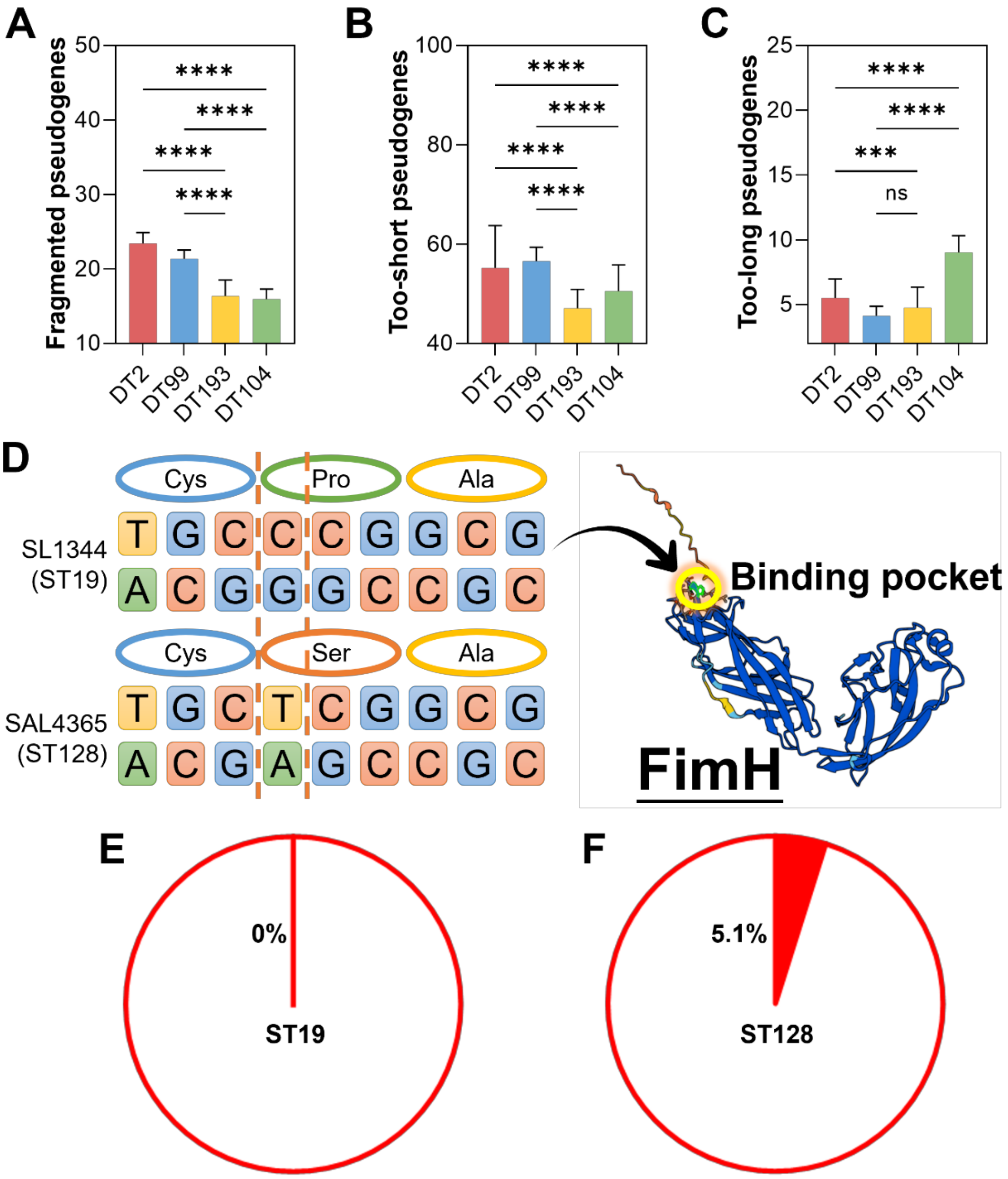

Supplement: S1 Fig — A. Predicted results of fragmented pseudogenes carriage. B. Predicted results of too-short pseudogenes carriage. C. Predicted results of too-long pseudogenes carriage. D. Schematic diagram of fimH mutation bases and the amino acid spatial locations where point mutations affect translation. E. Mutation rate in the STM ST19 population. F. Mutation rate in the STM ST128 population. (PDF) [file ppat.1012992.s001.pdf]
