# Supplementary material for: A genomic and phenotypic investigation of pigeon-adaptive Salmonella
Source: PLoS Pathog. 2025 Mar 17;21(3):e1012992. doi: 10.1371/journal.ppat.1012992 (PMC11957392; doi:10.1371/journal.ppat.1012992)

S2 Fig. *In vitro* phenotypic testing and chicken embryo infection results. A. Growth curve at 37°C under aerobic conditions. **B.** OD_{620} ratio changes in aerobic cultures at different temperatures. **C.** Motility diameter under different oxygen conditions. **D.** Biofilm formation capability at 37°C. **E.** Biofilm formation capability at 42°C. **F.** Chicken embryo survival curve.

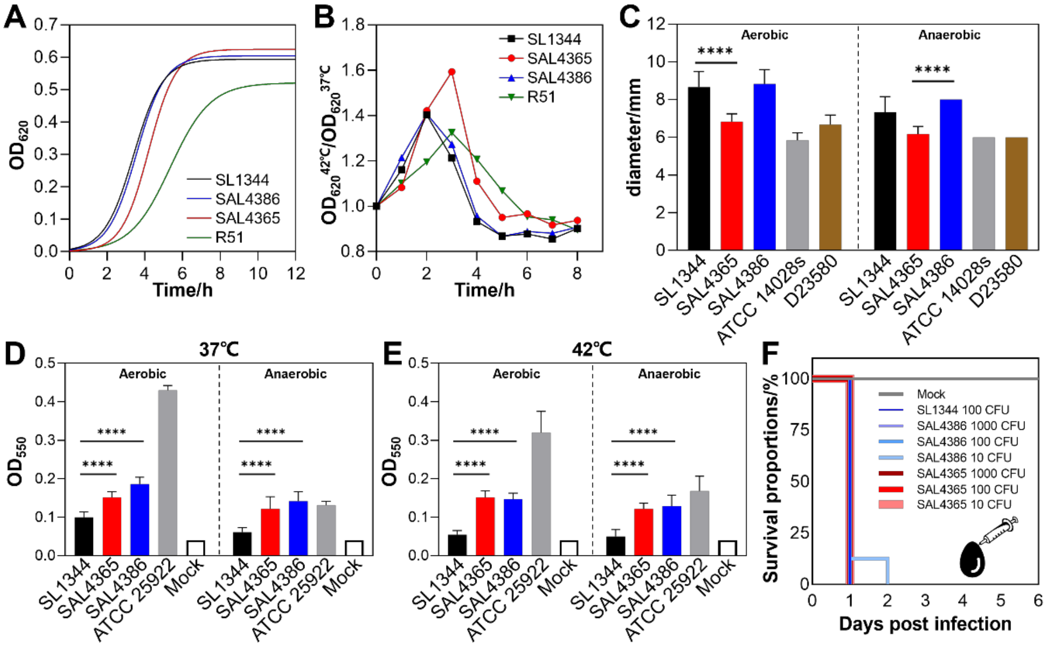

Supplement: S2 Fig — A. Growth curve at 37°C under aerobic conditions. B. OD620 ratio changes in aerobic cultures at different temperatures. C. Motility diameter under different oxygen conditions. D. Biofilm formation capability at 37°C. E. Biofilm formation capability at 42°C. F. Chicken embryo survival curve. (PDF) [file ppat.1012992.s002.pdf]
