# Supplementary material for: A genomic and phenotypic investigation of pigeon-adaptive Salmonella
Source: PLoS Pathog. 2025 Mar 17;21(3):e1012992. doi: 10.1371/journal.ppat.1012992 (PMC11957392; doi:10.1371/journal.ppat.1012992)
Supplement: S3 Fig — The red dashed lines connect the junction of the ileum and cecum in each group. In the SL1344 group, the liver presented with multiple pale lesions, a friable texture, and rounded edges. While hepatomegaly was also observed in the two poSTM experimental groups, the liver’s appearance in terms of color was similar to that of the Mock group, indicating minimal changes. The gallbladder and spleen in the SAL4365 and SAL4386 groups showed pathological enlargement, with sizes intermediate between those of the SL1344 and Mock groups. The colon of the SL1344 group mice was notably shorter than that of the poSTM experimental groups and exhibited numerous varicella-like nodules, with both the SL1344 and SAL4365 groups presenting with petechial hemorrhages. (PDF) [file ppat.1012992.s003.pdf]

S3 Fig. Pathological anatomy of enteritis model mice. The red dashed lines connect the junction of the ileum and cecum in each group. In the SL1344 group, the liver presented with multiple pale lesions, a friable texture, and rounded edges. While hepatomegaly was also observed in the two poSTM experimental groups, the liver's appearance in terms of color was similar to that of the Mock group, indicating minimal changes. The gallbladder and spleen in the SAL4365 and SAL4386 groups showed pathological enlargement, with sizes intermediate between those of the SL1344 and Mock groups. The colon of the SL1344 group mice was notably shorter than that of the poSTM experimental groups and exhibited numerous varicella-like nodules, with both the SL1344 and SAL4365 groups presenting with petechial hemorrhages.

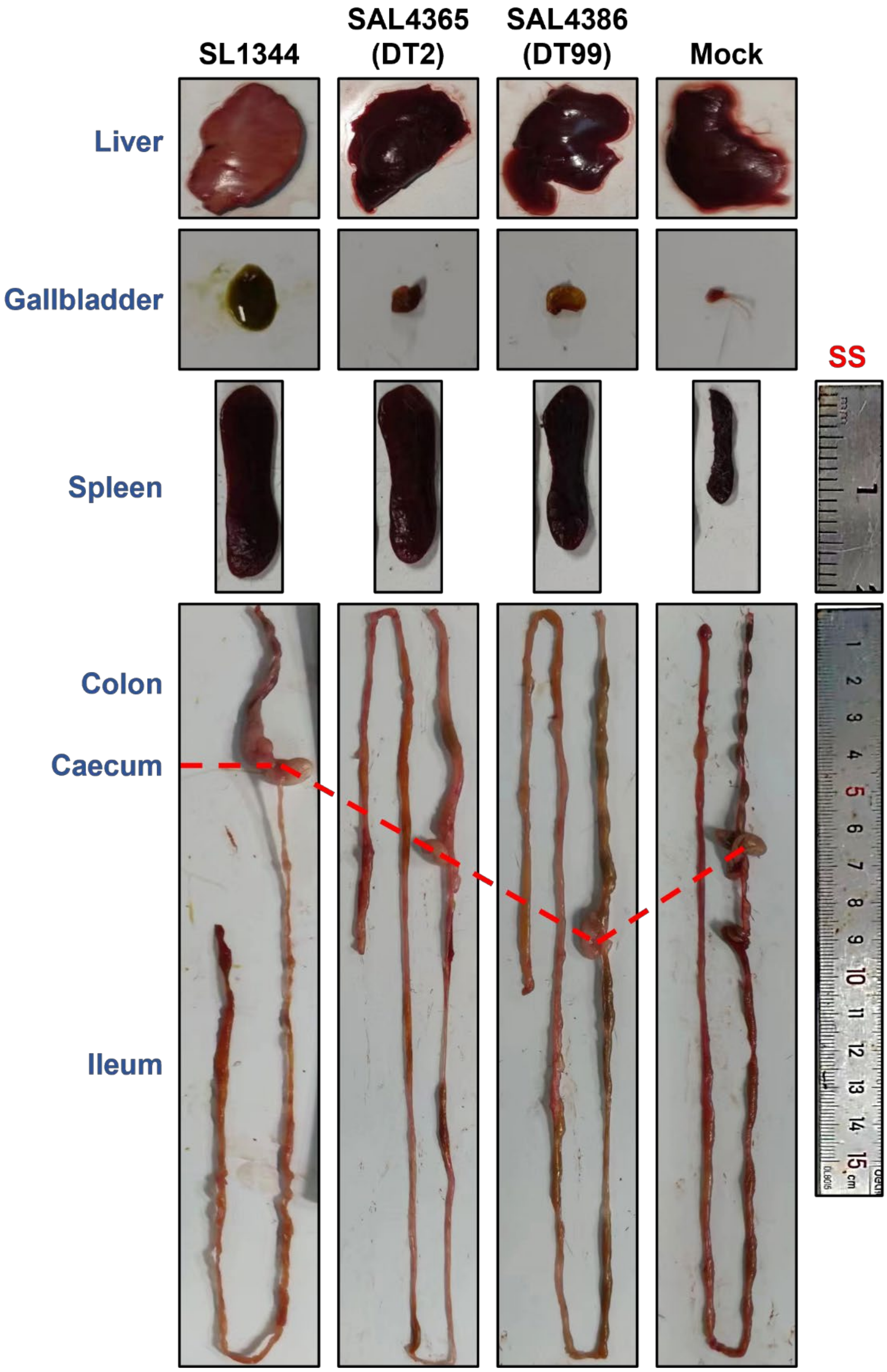
SL1344
SAL4365 (DT2)
SAL4386 (DT99)
Mock
Liver
Gallbladder
SS
Spleen
Colon
Caecum
Ileum
