# Supplementary material for: A genomic and phenotypic investigation of pigeon-adaptive Salmonella
Source: PLoS Pathog. 2025 Mar 17;21(3):e1012992. doi: 10.1371/journal.ppat.1012992 (PMC11957392; doi:10.1371/journal.ppat.1012992)

S4 Fig. Histopathological imaging of enteritis model mice tissue stained with HE (200×). The organs from the Mock group exhibited normal structures under the microscope, with clear cellular arrangements and no signs of edema or inflammatory cell infiltration. In liver sections, red arrows indicate vacuoles within hepatocytes, yellow arrows indicate inflammatory cell infiltration, and black arrows indicate hepatocyte necrosis; in spleen sections, yellow arrows indicate neutrophil infiltration, green arrows indicate iron deposition, blue arrows indicate an increased number of multinucleated macrophages, and red arrows indicate splenic cell necrosis. In intestinal sections, blue arrows indicate localized crypt structure loss, yellow arrows indicate inflammatory cell infiltration, black arrows indicate cell necrosis, and red arrows indicate localized mucosal epithelial cell ulceration and exposure of the lamina propria.

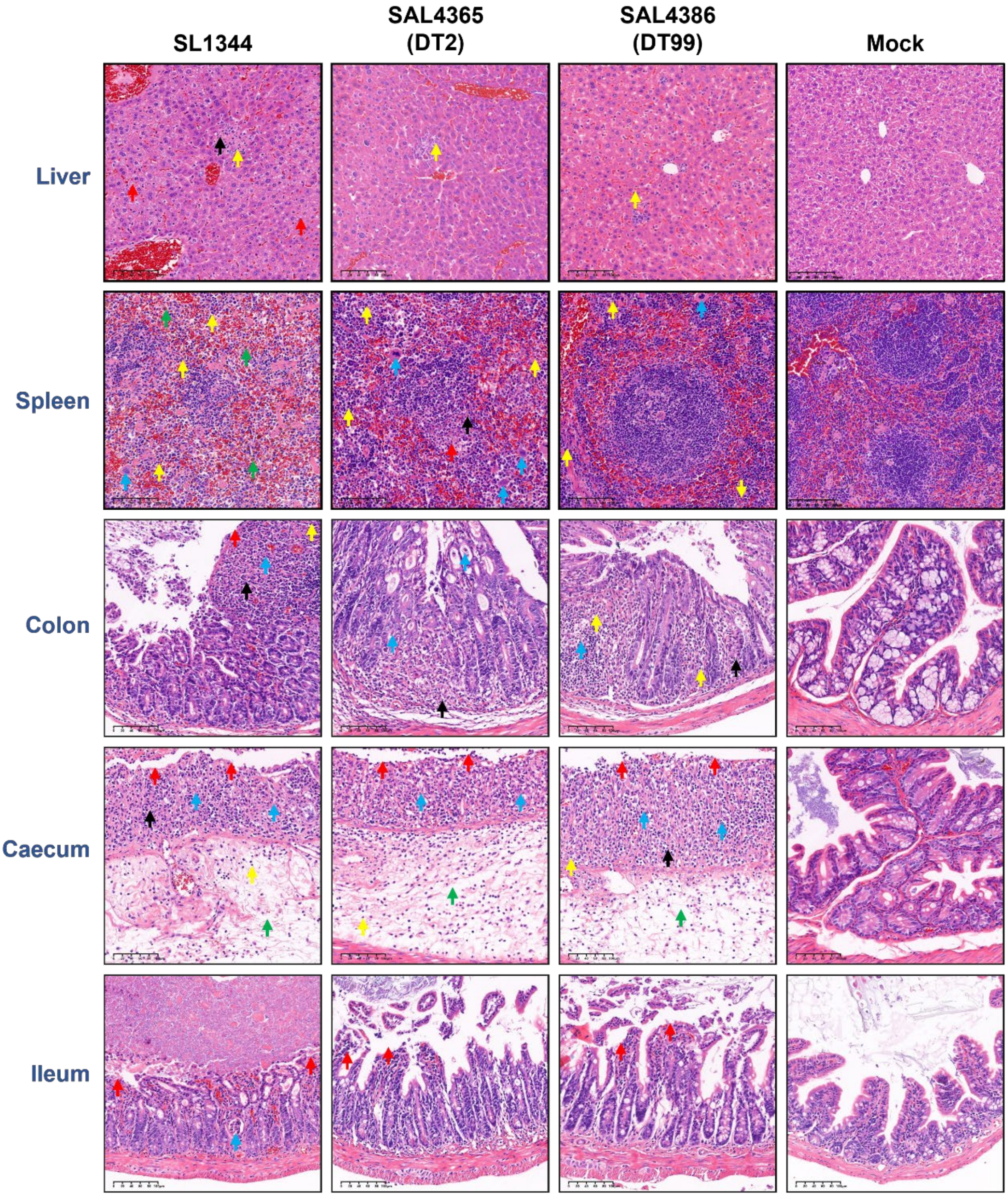

Supplement: S4 Fig — The organs from the Mock group exhibited normal structures under the microscope, with clear cellular arrangements and no signs of edema or inflammatory cell infiltration. In liver sections, red arrows indicate vacuoles within hepatocytes, yellow arrows indicate inflammatory cell infiltration, and black arrows indicate hepatocyte necrosis; in spleen sections, yellow arrows indicate neutrophil infiltration, green arrows indicate iron deposition, blue arrows indicate an increased number of multinucleated macrophages, and red arrows indicate splenic cell necrosis. In intestinal sections, blue arrows indicate localized crypt structure loss, yellow arrows indicate inflammatory cell infiltration, black arrows indicate cell necrosis, and red arrows indicate localized mucosal epithelial cell ulceration and exposure of the lamina propria. (PDF) [file ppat.1012992.s004.pdf]
