# Supplementary material for: A genomic and phenotypic investigation of pigeon-adaptive Salmonella
Source: PLoS Pathog. 2025 Mar 17;21(3):e1012992. doi: 10.1371/journal.ppat.1012992 (PMC11957392; doi:10.1371/journal.ppat.1012992)

**S5 Fig. Intestinal lesions of enteritis model mice and animal experimental design. A.** Ratio of villus height to crypt depth in the ileum. **B.** The mucosal layer thickness of the cecum. **C.** The mucosal layer thickness of the colon. **D.** Schedule of pathogenicity trials in enteritis model mice. **E.** Schedule of invasion trials in typhoid model mice.

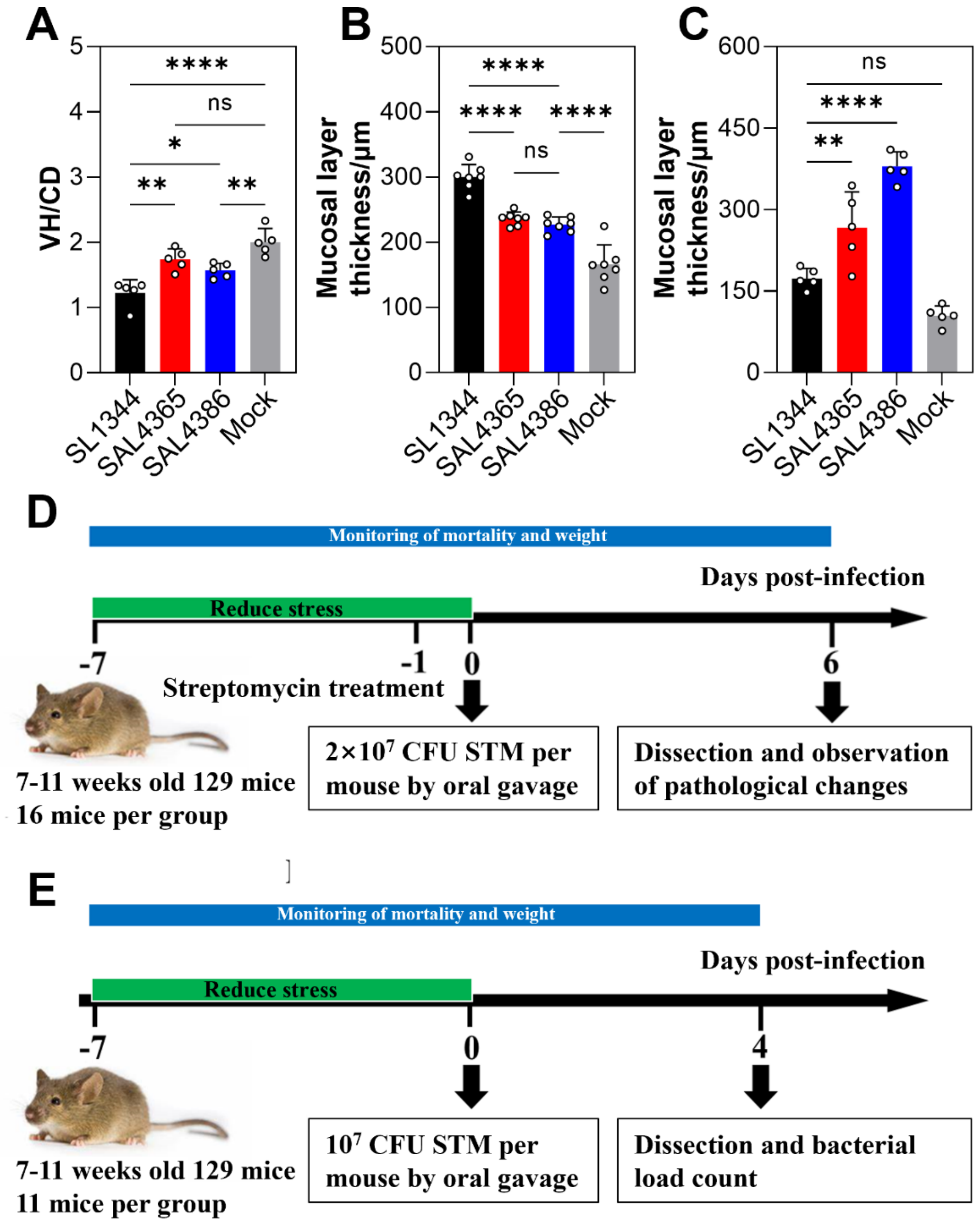

Supplement: S5 Fig — A. Ratio of villus height to crypt depth in the ileum. B. The mucosal layer thickness of the cecum. C. The mucosal layer thickness of the colon. D. Schedule of pathogenicity trials in enteritis model mice. E. Schedule of invasion trials in typhoid model mice. (PDF) [file ppat.1012992.s005.pdf]
